# Ethical consumption: why should we understand it as a social practice within a multilevel framework?

**DOI:** 10.12688/openreseurope.15069.1

**Published:** 2022-09-13

**Authors:** Sara Karimzadeh, Magnus Boström

**Affiliations:** 1 School of Humanities, Education and Social Sciences, Örebro University, Örebro, Örebro, 701 82, Sweden

**Keywords:** Ethical consumption practices, social practice theory, multilevel perspective, combination approach.

## Abstract

This article discusses the importance of a multilevel and intertwined understanding of ethical consumption given its conjunction with other social practices. Although the literature on ethical consumption is vast, the role of sociotechnical regimes including technological and cultural elements, infrastructure, market and regulation has been mainly overlooked in this literature. This may be so because ethical consumption practices that refer to other-oriented consumption practices are mainly considered in the view of the motivations and preferences of individual consumers. Due to the insufficiency of individualistic approaches to explain stimulators and inhibitors of ethical consumption, there might be other components in society to lead (un)ethical consumption decisions. Therefore, to avoid an oversimplified view of ethical consumption, this paper contributes with a theoretical discussion on combining social practice theory (SPT) with a multi-level perspective (MLP). Although the SPT is a very well-structured framework in consumption studies, the necessity of a combined approach concerns the often-insufficient attention paid to structural prerequisites of various consumption forms in social practice theories. By understanding ethical consumption practices according to a multi-level framework, the paper emphasizes the importance of structural factors at macro- and mesolevels. It also contributes attention to how ethical consumption grows due to dialectical processes between levels, showing that niche practices can, at the same time, both challenge and depend on existing regimes.

## 1. Introduction

Studying consumption as a social phenomenon that represents the fabric of society has opened a broad research space for social practice theory (SPT) in consumption studies in the recent decades. It is well recognized in SPT studies that we ought to lift the sight from the individual as a unit of analysis towards that of social practices in the socio-material surrounding. We agree with this but find that social structure and culture on ‘higher’ levels still often get insufficient attention. In line with this, several scholars have suggested combining the SPT and the multi-level perspective (MLP) to advance the understanding of consumption practices in general ^1^ ( el Bilali, 2019; Hargreaves *et al*., 2013; Hinrichs, 2014; Keller *et al*., 2022; McMeekin & Southerton, 2012; Spaargaren *et al*., 2012; Watson, 2012; Welch & Warde, 2015). This call can be extended to the study of ethical consumption, which is a partly overlapping phenomenon. Ethical consumption ^2^ refers to a set of consumption practices that are shaped by societal and environmental concerns related to green issues, workers’ rights and conditions, child labour, unfair trade, resource degradation, irresponsible marketing, animal testing, and oppressive regimes ( Berki-Kiss & Menrad, 2022; Carrigan *et al*., 2004; Carrington *et al*., 2010; De Pelsmacker *et al*., 2005; Freestone & McGoldrick, 2008; Huddart Kennedy *et al*., 2019; Wooliscroft *et al*., 2014). However, it remains contested how the representations of the phenomenon are formed and actualized in different societies and among different people. A great bulk of the literature on ethical consumption, particularly such inspired by marketing and psychological models, has tended to investigate the impacts of individual factors such as values, preferences and motives ( de Pelsmacker *et al*., 2005; Freestone & McGoldrick, 2008; Shove, 2010; and Zaikauskaitė *et al*., 2022). For example, in a recent study, Berki-Kiss & Menrad (2022), suggest that consumer knowledge, attitude and emotions are significant factors to push consumers to purchase agricultural non-food (fairtrade cut roses) ethical products ( Berki-Kiss & Menrad, 2022). Likewise, Carrington *et al*. (2010) through investigating research models on consumption behaviour indicate that ethical purchasing intentions are mainly driven by factors such as personal values, moral norms, mental processes and internal ethics ( Carrington *et al*., 2010). Can we claim that these factors are merely individual factors? Can we see consumers as autonomous and independent actors in social structures? However, the remarkable “intention-behaviour gap” among ethical consumers’ ( Belk *et al*., 2005; Carrigan & Attalla, 2001; Zaikauskaitė *et al*., 2022) indicates there might be either “various constraints” in society and “competing demands” to hamper consumers from acting ethically ( Carrington *et al*., 2010). In order to avoid oversimplifying our understanding of consumer behaviour to linear psychological models ( Bagozzi, 2000) (like the Theory of Planned Behaviour (TPB); and Value-Belief-Norm (VBN)), it is important to understand ethical consumption as context-dependent ( Zaikauskaitė *et al*., 2022) that is influenced by social structures, technical infrastructures, available knowledge, culture and social norms.

Social practice theory is a theoretical upgrade in consumption studies that would be useful in the study of ethical consumption and filling gaps in the scholarship. This theory introduces a proper framework to consumption studies by making interconnections between the roles of material, meaning and competencies in the creation of practices ( Gram-Hanssen, 2021). It argues that people’s actions are influenced by their socio-material context, what they understand and receive from their environment, as well as their obligation toward others ( Heisserer & Rau, 2017; Rinkinen *et al*., 2020). Although by emphasizing mainly everyday life, the SPT has opened a broad theoretical area to consumption studies, key analytical critiques concern its insufficient attention paid to macro- or structural pre-requisite of various consumption forms ( Keller *et al*., 2022; Greene, 2018; Welch, 2020), and “supply side dynamics (like firms, innovation systems, technical capabilities” ( Geels *et al*., 2015: p. 10). Also, the power issue and role of macro-scale social inequalities in the formation of daily routines are almost absent in its endeavours to theorize consumption practices ( Geels *et al*., 2015; van Kesteren & Evans, 2020). A greater theoretical lens that gives a broader picture of the formation of ethical consumption will enrich our sociological understanding of the systemic and structural factors that delimit ethical consumption agency or (re)define it according to the specific sociotechnical context. It also helps to shed light on the phenomenon from different angles. The need is even more urgent if we want to address, for instance, firstly the very different conditions for ethical consumption practices in different geographical contexts, and secondly, newly established forms of consumption practices that are mainly associated with the structural attributes of the society in question. Therefore, understanding ethical consumption as practices that come about in multi-level frameworks, this paper provides a deeper conceptual insight into social (im)possibilities of the formation of ethical consumption. It also contributes attention to how ethical consumption grows due to dialectical processes between levels, which, as well, helps to avoid the tendency of overly individualized perspectives on ethical consumption.

The next section, by introducing the SPT and MLPs, briefly discusses how these frameworks can mutually enrich a developed analytical perspective regarding ethical consumption. Section three provides examples of ethical consumption in different sociotechnical regimes across the world. Section four discusses the configuration of ethical consumption within social regimes and also the dialectics between the levels in forming ethical consumption practices. This section is followed by the conclusion.

## 2. SPT and MLP: a combined perspective to explain the routinization and upscaling process of newly emerged consumption practices

By following insights gained from MLP ( Geels, 2002) and recent scholarship, ethical consumption can be considered as a complex multilevel phenomenon “which involves more than an individual’s behaviour and practices” ( Boström *et al*., 2019: p. 4). Applying a multi-level analysis frame is a way to understand how practices are formed within a pre-given set of heterogeneous elements such as norms, conventions, infrastructures, knowledge, technology and other structural conditions like systems of provision associated with consumption practices in different levels of society. Despite the possible conflicts and tensions, practices evolve due to interdependencies and interconnections among these elements ( Boström *et al*., 2019) and reflect in social practices like consumption.

To understand social practices as a combination of elements, a useful and much-cited framework in consumption studies is the one suggested by Shove *et al.* (2012). They argue that a specific practice occurs through specific connections between three components: 1) materials and technology (artefacts, infrastructures, and hardware); 2) meanings (images, understandings, feelings, mental activities, emotions and motivational knowledge); and 3) competencies and skills (background knowledge, know-how, general understanding) ( Shove *et al*., 2012). To apply this combination to the ethical consumption context, the alignment of the meaning of ethical consumption (a culture of caring for the environment, community and society; that is, caring attitudes), competencies (how to do and develop this protection) and material (infrastructures and technologies that make this opportunity available for people, including market products) navigate this practice. Co-evolution between meaning, material and competencies may lead to a transition in (or transformation of) practices. In the absence of one of these elements, the ethical consumption practice would not be able to make a room for itself in society. In other words, an external intervention to bring in new technology (material) or spread new knowledge and education (competence), as well as facilitating niche innovations or communities of practitioners that try to change conventions and norms (meanings), may lead to a social shift that eventually is followed up by practice change.

According to Geels (2002), the MLP provides a multi-layer analysis that sees sustainable practices as the result of the dialectic interaction between three levels of the micro (niche), meso (sociotechnical regimes), and macro (landscape). In this framework, the micro level refers to the spaces where innovative activities led by niches take place on small scales. The meso level, consisting of regimes, refers to the existing sociotechnical systems including the network of actors and social groups, rules and related technical and material elements. Finally, the macro level or landscape includes a set of external heterogeneous events and trends such as cultural changes, macroeconomic trends, wars and crises, pandemics like COVID-19, climate change, *etc*. The MLP locates technological and organizational innovations at the center of its analysis and argues that broader sociotechnical, economic and political contexts create more or less favourable circumstances for such innovation ( McMeekin & Southerton, 2012). In this perspective, regimes are home to incremental innovations whereas radical innovations are generated in niches. However, change of dominant practices rarely happen without a level of co-evolution of all three levels.

However, even though practice theorists do speak of how practices are embedded in socio-material arrangements in society, there is arguably insufficient attention paid on larger scales. Welch & Warde (2015; 12–13) raise several criticisms of the SPT, one of those criticisms is particularly the focus of this study: social practice theories fail in relating the *“minutiae of everyday performances of practices and the macro-institutional context*”. Considering niche dynamics as fluid novelties that are created at the micro-level and which might be obstructed by incumbent regimes ( Keller *et al*., 2022), we argue that more attention needs to pay on how dispersed practices are able to expand to a wider range through supportive mechanisms at the meso-level ( *i.e*. civic groups, environmental groups) and moreover shape and reshape the macro-level setting (while at the same time being shaped by macro-level factors). Furthermore, by locating ethical consumption practices in the social context and considering their interconnectivity to other practices ( *e.g*. food practices, cleaning practices, commuting, *etc*.), we argue there is a need to pay attention to different forms and perceptions of the phenomenon that are created in accordance with the specific contextual situations (geography, sector-wise). Broad contextual factors include material and technological development ( Heisserer & Rau, 2017; Warde, 2014), economic welfare ( Evans, 2020), the system of provision ( Fine & Bayliss, 2022) and political democratic culture and discourse ( Gundelach, 2020; Portilho & Micheletti, 2019). The advantage of the combination of the MLP and SPT in this context is to theorize in a deeper sense the structural conditions in terms of market, suppliers and producers’ networks, policies, technology, science and knowledge (MLP), and bring further attention to cultural features such as the importance of meaning and competencies embedded in the immediate social and physical context (SPT).

While the SPT pays insufficient attention to macro-institutional elements, the MLP could be criticized for over-emphasizing them by prioritizing the role of sociotechnical regimes. A critique of the MLP is that it appears to propose a technical-based, mechanistic and over-determined view ( Geels *et al.*, 2015; Hinrichs, 2014). However, we argue it is important with a perspective that recognizes that inertia is built into the system, and where nested regimes are stabilized and rarely undergo transformation. Yet, change is not deemed impossible, but we need to be more aware that moving towards sustainable/ethical consumption requires, in addition to technological changes, changes in consumer practices, cultural meanings, infrastructures, and economic practices as well ( Geels, 2019). “[T]echnology of itself, has no power, does nothing” ( Geels, 2002: 1257) and therefore the significance of human agency and organizational structure in moving towards societal transformation must be considered.

## 3. Ethical consumption in different contexts

By broadly viewing ethical consumption in different contexts, it becomes even clearer how both ‘landscape’ and ‘sociotechnical regimes’ shape conditions for (the organizing of) ethical consumption practices. It is mainly emphasized that ethical consumers “talk” through their consumption choices (read power) to buy or refrain from purchasing goods or services which do or do not meet their ethical criteria when it comes to social and/or environmental standards ( de Pelsmacker *et al*., 2005). Nevertheless, it is unclear whether this seemingly simple definition is feasible in every socio-political and/or sociotechnical regime.

Figures of ethical consumption in the western contexts reveal that the extent of ethical/political consumption in comparison to both non-Western Europe and non-European contexts is very high ( de Moor & Balsiger, 2019; Koos, 2012). In addition to the explanations raised by the TPB ( Ajzen, 1991) and the VBN ( Stern, 2000) that generally study the significance of individual factors ( *i.e.* consumers’ values, attitudes, subjective norms and knowledge), other plausible explanations, from sociological perspectives, relate to the relatively high level of welfare in the region along with the emergence of the post-material values. Moreover, well-educated populations and free access to means of communication are two effective factors according to Boström *et al*. (2019). This finding is in line with the Jacobsen & Dulsrud (2007) report that ethical consumption emerges in a more liberal world economy where new consumption forms are supported by non-governmental organizations, promote sovereign consumers’ ideas and provide alternative products and businesses ( Jacobsen & Dulsrud, 2007). Nevertheless, such patterns do not mean that ethical consumption is formed in western culture exclusively. Comparative studies reveal the importance of historical and geographical factors in the formation of various ethical consumption patterns among different populations (see Boström *et al*., 2019).

There are some studies covering the phenomena in different geographies, and, interestingly, they are uncovering different interpretations of ethical consumption among different societies. Principles like justice, fairness, environmental protection, social solidarity and sustainability more broadly can appear in different ways and shapes. Hence, ethical consumption is a phenomenon that can be formed, realized, interpreted and demonstrated differently thanks to time, place, and circumstances. For example, in Africa, political/ethical consumption has been a response to the corrupted social and political system while in the MENA ^3^ region, consumption practices are greatly influenced by rapid economic transition, social development, democratic uprising, wars, political violence and religious contradictions ( Oosterveer *et al*., 2019). Moreover, Hughes *et al.* (2015) brought up that in the context of Africa, “localized expression of ethical consumption” is essential in transformations on the supply-side ( Hughes *et al*., 2015). Oosterveer *et al*. (2019) yet highlight the significance of the insufficient (social) base to explain the absence of ethical/political consumption in African and MENA countries. As regards Latin America, Portilho & Micheletti (2019) discussed the role of social movement struggles to push for a stronger state to regulate the market and revealed the significance of public policies and state regulation in promoting sustainable products and ethical consumption ( Portilho & Micheletti, 2019). In Thailand, there has been rapid industrialization, which has provided opportunities for social movements to encourage Thai consumers to influence consumption-related policymaking and participate in discussions, for instance via social media, about changed social practices ( Kantamaturapoj *et al*., 2019). In China, ethical/political consumerism “can only be understood in a broader context of institutional changes over the past decades” ( Lei *et al*., 2019: 598). Due to the governmental measures that delimit consumers’ choices, political/ethical consumption cannot be similar to the phenomenon in Western and Northern countries ( Lei *et al*., 2019) and requires more creative initiatives in social systems that provide a limited assortment of ethically framed goods and services ( de Moor & Balsiger, 2019; Koos, 2012). Furthermore, research indicates that a lack of information on companies’ practices is a key obstacle to socially responsible consumption ( Toti & Moulins, 2016). Corporate social responsibility (CSR) is also a key factor in the promotion of ethical consumption ( Sudbury-Riley & Kohlbacher, 2016) due to its positive role in encouraging producers to produce under ethical standards (even though sometimes targeted for greenwashing and watered-down ethical standards) and facilitating choice infrastructures for consumers. However, the main focus here is associated with more infrastructural supply-side factors. In a social structure with transparent regulations and laws regarding the social responsibilities of cooperation, producers are obliged to reflect the interests of stakeholders like employees, investors and the environment in their actions and policies and it directs, in turn, consumers to ethical decisions ( Adams & Zutshi, 2004). Whereby corporations' policy to maximise their interest *via* CSR facilitates ethical decisions among consumers. From a multi-level perspective, in the lack of an efficient regulatory regime as well as technical and cultural support, CSR will prove insufficient to meet ethical consumers’ demands from the market.

Based on the knowledge of ethical consumption scholarship obtained from different social contexts, it becomes clear that the phenomenon must be considered as an intertwined phenomenon with sociotechnical regimes that shape market structures, laws and regulations, infrastructure and materials as well as culture and norms. Markets, governance structure and the settings of everyday life frame consumers’ decisions and practices ( Jacobsen & Dulsrud, 2007). Therefore, niche novelties regarding ethical consumption are created in and influenced by the given situation ( *i.e*., available knowledge, system of provision, alternative markets and democratic governances). Therefore, studying ethical consumption practices in the light of the multilevel perspective brings attention to how everyday practices are formed and changed within particular sociotechnical regimes. It should also be kept in mind that due to the niche’s capacity to fuel changes, any corporation or conflict between the elements of the micro and meso level can act as an assistance or obstacle to expanding niche novelties to more routinized and integrated practices.

## 4. Configuration of ethical consumption within multi-level interactions

When it comes to ethical consumption, niche innovations are often recognized as resistance to mainstream consumption and markets. Examples include asking for fair-waged, organic, and cruelty-free cosmetic and clothing products (buycotting), creating household initiatives to produce less waste or consume less energy, supporting local agriculture and products, and refusing from buying products or services with unfair work conditions or unclear production and distributing processes (boycotting). Some of these initiatives have become mainstream in some western societies, but appear radical, confrontative and/or illegal in several other contexts. Furthermore, decreasing the volumes of consumption, using second-hand stuff, and trying to challenge public attitudes by criticizing excessive consumerist lifestyles or encouraging other people to support eco-friendly and/or socio-friendly products (discursive ethical consumption) are other examples of mainly niche ethical consumption performances. These niche efforts are linked with efforts to, in the long run, reconfigure existing regimes ( Hargreaves *et al*., 2013). This indicates that the embodiment of ethical initiatives in everyday life is conducted in sets of interdependencies within the different systems of provision and practices like eating, commuting, and cleaning ( Nicolini, 2009).

Niche practices, like various trends in ethical consumption, reflect perceived deficiencies in regimes and landscapes. Nevertheless, they are configured and developed within existing regimes’ capacities. Greene indicates that “a complex web of contextual processes including technological change, economic transitions and planning policies” are significant factors in the formation of consumption practices ( Greene, 2018: 1). Hence, niche ethical consumption practices are also contingent upon sociotechnical regimes in terms of infrastructures, cultural meanings, policies and conventions, markets, knowledge systems and (semi-)dominant discourse. Therefore, perceiving ethical consumption forms like supporting fairtrade or fair paid products, waste segregation, collaborative consumption, ethical lifestyle, *etc*. as autonomous and independent novelties which are led only by individuals’ decisions and preferences misleads us in understanding the whole story of ethical consumption subject. Oosterveer *et al*. (2019: 135) indicate that “ethical consumption goes beyond individualistic choices” and relates to sociotechnical systems ( *e.g*. institutions, products, infrastructure, services, social relationships and objects). This means that although niche innovations germinate separately among detached actors they are still being created inside the social setting and a level of regime support is necessary for their upscaling. For example, a regime may support a niche activity such as a boycott campaign either by providing space for democratic expression and media space for its promotion or by declaring new regulations and policies regarding the production processes ^4^. Furthermore, social movement campaigns can take advantage of the social and democratic space and support that is given to their claims and objectives ( Forno, 2019). All societies do not provide such opportunities for niches to pursue their novel objective on a bigger scale, but this knowledge is too often taken for granted in theorizing about the opportunities for niche practices to grow.

Geels argues that niche innovations will not spread widely unless external landscape developments create pressures on the regime, destabilize it, and then create space for novelties ( Geels, 2005). Therefore, a synergetic mode between macro factors and micro initiatives facilitates the deconstruction of the nested regimes and the construction of new ones. For instance, a landscape change as the COVID-19 pandemic, which caused disruption of many conventional practices, has been seen to provide a window of opportunity for more “mindful consumption”, in which issues of health and environmental protection are considered more among broad segments of consumers ( Boström, 2021; Echegaray *et al*., 2021). However, landscape-level factors can appear as obstacles as well. For example, poverty in all its forms doesn’t allow deprived people to (re)form their consumption practices aligned to the routinized consumption practices or niche novelties. A number of studies moreover indicate that “privileged groups” in terms of higher-income levels and more education are more likely to be ethical/political consumers ( *e.g*. Salonen, 2021).

Sociotechnical regimes might also have two edges: supportive or deterrent or a mix of both. In this vein, we have examples that many niche dynamics have been oppressed or marginalized in many social regimes so far, or at least have been unable to scale up. For example, Marsden (2013) points out some food niche initiatives that are marginalized in the nested food governance regimes and therefore, proposes the necessity of policy spaces to facilitate niche paths through multilevel regulations ( Marsden, 2013). Kesteren & Evans (2020) refer to how socio-economic inequalities through delimiting the capacity of practitioners affect cooking practice in terms of competencies, materials and meaning. Therefore, structural conditions in terms of top-down governance, infrastructures, upstream policies, institutions, actor networks, capabilities and resources can be both enabling and constraining for transition ( Boström, 2020; Slingerland & Schut, 2014). On the level of sociotechnical regimes, their role to develop ethical consumption on a large scale relates largely to existing structures/relations of power. This with regards to resource distribution, opportunities to pose new regulations, controlling markets, financial flows and international trade relations, investment in new technologies and materials, controlling media and making room for public debate, as well as providing public education. All these factors can prevent or enable the normalizing of novel ethical consumption practices.

The above discussion shows there is a dialectical process between the levels. That is, to normalize a new practice both niches and regimes need each other, facilitated by broad landscape movements in the early stage, knowledge and information that consumers obtain from formal or informal learning environments are critical to developing niche ethical consumption practices. The perceived meaning of ethical consumption among niches, the skills and competencies that they obtain being an ethical consumer and the materials that are given to assist them in their ethical consumption practices, are all generated in part through the sociotechnical regimes that surround them. While this implicates the interdependencies of ethical consumption practices on one another, it also indicates how they can embed in particular sociotechnical regimes ( Keller *et al*., 2022). This reading also challenges the psychological and marketing approaches that advocate consumer sovereignty to make wide-range changes in unsustainable consumption cultures.

In addition to technical and infrastructural facilitators, the meaning (objectives, images and perceptions) that consumers realize from ethical consumption influences their performances and can spread in social networks. In a social structure that promotes awareness and knowledge regarding consumption-related issues, in different ways (through media debates, civil society organizations, alternative markets and products), practitioners are more likely to broadly engage in ethical consumption and have better know-how skills to implement it. Geels *et al.* (2015) argue that dispersed niche practices are less likely to be developed to the broader scale of meso-level unless taking advantage of broad learning processes and social network building ( Geels *et al*., 2015). Such processes can be referred to as a sort of teleoaffective understanding that makes ethical consumption meaningful. Teleoaffective understanding integrates practices through goals and the meanings that practitioners perceive and carry out. It means that through practical intelligibility, practitioners carry on actions that make sense to them ( Heisserer & Rau, 2017) and are doable. Desires, beliefs and expectations are examples of teleoaffectivity ( Schatzki, 2001) that help to create a general understanding of the subject. In the ethical consumption context, a common understanding among practitioners may organize them into an overarching cultural formation. In the lack of such understanding (culture, teloaffectivity), this phenomenon can only remain as a dispersed practice among individual practitioners and cannot spread to a large scale.

## 5. Conclusion

This article discusses the importance of a multilevel and intertwined understanding of ethical consumption given its conjunction with other social practices. Social Practice theory, by understanding consumption as a multifaceted practice and interconnected to other social practices offers a promising perspective to studying consumption practices. However, this article argues that although this is a useful approach to theorizing consumption practices, it is not sufficient to shed light on all aspects of a phenomenon such as ethical consumption, which is a very context-related concept. We emphasized that practices are only able to be fully accomplished through interconnected (elements in) sociotechnical regimes, also facilitated by landscape patterns, and therefore are needed to be analysed by applying a form of multilevel perspective. Even if the role of meso-factors is recognized in various studies, we believe there is a tendency for them to be too much taken for granted. The different extent, shapes, and ways of ethical consumption practices throughout the world illustrate the importance of not bracketing factors relating to ‘landscape’ and ‘sociotechnical regimes’. We ought to detach ethical consumption from an overly individualistic view, locate it as embedded in social life and social practices, and recognize how the practices are developed and organized through dialectical processes within a multi-level framework. In this way, we can better grasp the conditions for organizing and upscaling ethical consumption practices in different sectors and countries around the world. Attention to such multi-level dynamics shows that new ethical consumption practices can both challenge and rely on existing regimes at the same time. Further studies are needed to deconstruct the phenomenon within different levels of the social system in order to provide empirical knowledge to support the proposed idea.

## Ethics and consent

Ethical approval and consent were not required.

## Data Availability

No underlying data are associated with this article.

## Footnotes

^1^ About similarities and differences between the SPT and MLP and the advantages of co-employing them in consumption-related topics see Keller *et al*. (2022)

^2^ similar concept is political consumption. See The Oxford Handbook of Political Consumerism Edited by Boström *et al*. (2019).

^3^ Middle East and North Africa

^4^ Similar to what happened against the Apartheid regime in South Africa, there are also examples of international boycott campaigns that have mobilized people through market-based strategies to act against oppressive regimes (for more examples see Boström *et al*., 2019).
