# Supplementary material for: Ethical consumption: why should we understand it as a social practice within a multilevel framework?
Source: Open Res Eur. 2022 Dec 21;2:109. Originally published 2022 Sep 13. [Version 2] doi: 10.12688/openreseurope.15069.2 (PMC10445865; doi:10.12688/openreseurope.15069.2)
Supplement: Peer review history

# Peer review: Reviewer response for version 2

**DOI:** 10.21956/openreseurope.16649.r30498

**Published:** 2023-01-23

**Authors:** Kirsten Gram-Hanssen, Line Kryger Aagaard

**Affiliations:** 1 Department of the Built Environment, Aalborg University Copenhagen, Copenhagen, Denmark

With the additions and revisions made, I find the paper ready for indexing.

Is the argument persuasive and supported by appropriate evidence?

Partly

Is the work clearly and cogently presented?

Yes

Is the topic of the essay discussed accurately in the context of the current literature?

Partly

Does the essay contribute to the cultural, historical, social understanding of the field?

Yes

Reviewer Expertise:

consumption and theories of practice

We confirm that we have read this submission and believe that we have an appropriate level of expertise to confirm that it is of an acceptable scientific standard.

# Peer review: Reviewer response for version 2

**DOI:** 10.21956/openreseurope.16649.r30499

**Published:** 2023-01-11

**Authors:** Philip Balsiger

**Affiliations:** 1 University of Neuchâtel, Neuchâtel, Switzerland

No further comments.

Is the argument persuasive and supported by appropriate evidence?

Partly

Is the work clearly and cogently presented?

Yes

Is the topic of the essay discussed accurately in the context of the current literature?

Partly

Does the essay contribute to the cultural, historical, social understanding of the field?

Yes

Reviewer Expertise:

Ethical consumption, social movement studies, economic sociology

I confirm that I have read this submission and believe that I have an appropriate level of expertise to confirm that it is of an acceptable scientific standard.

# Peer review: Reviewer response for version 1

**DOI:** 10.21956/openreseurope.16296.r30122

**Published:** 2022-11-04

**Authors:** Philip Balsiger

**Affiliations:** 1 University of Neuchâtel, Neuchâtel, Switzerland

This article proposes and offers a critique of studies of ethical consumption from an individual perspective and proposes to enhance understandings of ethical consumption by studying it from a social practice and multi-level perspective. It points at an important short-coming of the literature and presents interesting examples to advocate for the heuristic contribution of SPT and MLP for analyzing ethical consumption, in particular by pointing out the role of socio-technical regimes. I suggest four points for improvement:

1. First, some improvements could be made in the presentation of the relevant literature. While the authors present evidence for the prominence of studies on ethical consumption using an individual (and often psychological) perspective, there are studies in this realm that are more attentive to context (without using SPT or MLP). For instance, scholars putting forward the importance of social movements as a meso-level context, or the significance of supply. This is discussed widely in de Moor & Balsiger (2019).
2. A second critique concerns section 3 of the article (Ethical consumption in different contexts). This section does show studies from geographically dispersed contexts showing how ethical consumption often follows different logics and functions quite differently. However, the impression appears that sometimes, the differences pointed out are more due to the scholarly interests and foci of the different scholars. For instance, the role of public policies in driving ethical consumption has also been highlighted for European contexts (see Dubuisson-Quellier *et al.*, 2016 ^1^ and Wahlen and Dubuisson-Quellier, 2018 ^2^) – not just in Latin America.
3. Third, the paper is quite uneven with regard to its dual objective of advocating for the usefulness of SPT *and *MLP. It does present both of them in part 2 (without however discussing their compatibility), but then in the more illustrative parts (especially section 4), the discussion concerns almost exclusively the multi-level perspective. It might be better to focus the paper solely on the MLP, since this is already a little bit the case in terms of focus, and since it is theoretically quite dense otherwise.
4. Fourth, in terms of the integration of the MLP in the study of ethical consumption, the paper makes some very valuable suggestions of the potential contributions of this. These could be presented more systematically. There is in particular the double question of a) how socio-technical regimes constrain and fashion the development of ethical consumption practices, and b) how ethical consumption niches can spread and change landscapes and regimes.

Finally, and building on the above point, since the paper advocates for the use of SPT and MLP in the study of ethical consumption, it might be useful to have some kind of a research agenda at the end. To suggest a number of research avenues and questions such a theoretical approach implies.

Is the argument persuasive and supported by appropriate evidence?

Partly

Is the work clearly and cogently presented?

Yes

Is the topic of the essay discussed accurately in the context of the current literature?

Partly

Does the essay contribute to the cultural, historical, social understanding of the field?

Yes

Reviewer Expertise:

Ethical consumption, social movement studies, economic sociology

I confirm that I have read this submission and believe that I have an appropriate level of expertise to confirm that it is of an acceptable scientific standard, however I have significant reservations, as outlined above.

## References

[1] : Gouverner les conduites. *Presses de Sciences Po* .2016; Reference source

[2] : Consumption Governance Toward More Sustainable Consumption. *Journal of Family & Consumer Sciences* .2018;110(1) : 10.14307/JFCS110.1.7 7-12 10.14307/JFCS110.1.7

# Response

**Published:** 2022-11-29

**Authors:** Sara Karimzadeh

**Affiliations:** Örebro University, Sweden

Dear Philip, Thank you very much for your constructive comments. In the following, we briefly describe changes that we have made in the paper according to your comments.

 This article proposes and offers a critique of studies of ethical consumption from an individual perspective and proposes to enhance understanding of ethical consumption by studying it from a social practice and multi-level perspective. It points at an important short-coming of the literature and presents interesting examples to advocate for the heuristic contribution of SPT and MLP for analyzing ethical consumption, in particular by pointing out the role of socio-technical regimes. I suggest four points for improvement:

- First, some improvements could be made in the presentation of the relevant literature. While the authors present evidence for the prominence of studies on ethical consumption using an individual (and often psychological) perspective, there are studies in this realm that are more attentive to context (without using SPT or MLP). For instance, scholars putting forward the importance of social movements as a meso-level context, or the significance of supply. This is discussed widely in de Moor & Balsiger (2019).

Response: We agree social movements are very important as well as relevant to highlight more, and we addressed this comment in the introduction section (in the last part of the first paragraph) and also made some changes in section two.

  

- A second critique concerns section 3 of the article (Ethical consumption in different contexts). This section does show studies from geographically dispersed contexts showing how ethical consumption often follows different logics and functions quite differently. However, the impression appears that sometimes, the differences pointed out are more due to the scholarly interests and foci of the different scholars. For instance, the role of public policies in driving ethical consumption has also been highlighted for European contexts (see Dubuisson-Quellier *et al.*, 2016 ^1^ and Wahlen and Dubuisson-Quellier, 2018 ^2^ ) – not just in Latin America.

Response: We understand the previous version could bring this impression, and have, in response to this comment, developed section 3 (mainly the second paragraph) by referring to the mentioned studies and two more studies as well.

  

- Third, the paper is quite uneven with regard to its dual objective of advocating for the usefulness of SPT *and *MLP. It does present both of them in part 2 (without however discussing their compatibility), but then in the more illustrative parts (especially section 4), the discussion concerns almost exclusively the multi-level perspective. It might be better to focus the paper solely on the MLP, since this is already a little bit the case in terms of focus, and since it is theoretically quite dense otherwise.

Response: We chose to keep the logic of the structure but added pieces on SPT-related work in accordance with Hanssen's comment number 1 and 2.

  

- Fourth, in terms of the integration of the MLP in the study of ethical consumption, the paper makes some very valuable suggestions of the potential contributions of this. These could be presented more systematically. There is in particular the double question of a) how socio-technical regimes constrain and fashion the development of ethical consumption practices, and b) how ethical consumption niches can spread and change landscapes and regimes.

Response: We agree there is a double question, but they are at the same time always connected. Therefore, we have tried to make some clarifications and added examples in section four (to indicate both Qs), but kept the general structure of the argumentation. This dynamic is also itself a key topic for future research, which we tried to indicate in the concluding section.  

- Finally, and building on the above point, since the paper advocates for the use of SPT and MLP in the study of ethical consumption, it might be useful to have some kind of a research agenda at the end. To suggest a number of research avenues and questions such a theoretical approach implies.

Response: According to our arguments in the paper, we suggested some ideas at the end of the paper.

# Peer review: Reviewer response for version 1

**DOI:** 10.21956/openreseurope.16296.r30091

**Published:** 2022-10-10

**Authors:** Kirsten Gram-Hanssen, Line Kryger Aagaard

**Affiliations:** 1 Department of the Built Environment, Aalborg University Copenhagen, Copenhagen, Denmark

Thank you for this opportunity to review an interesting and timely contribution on the theoretical foundation for understanding ethical consumption. As the paper writes, theories of practice have been utilized for understanding consumption broadly, and more recently also specifically for understanding ethical consumption. However, as the paper also points out, most consumer studies so far based on theories of practice have been reluctant in studying overarching cultures and structures of consumer practices. For this reason, this paper suggests combining theories of practice, or social practice theory (SPT) with multi-level perspectives (MLP) theories, which we agree is a relevant approach to take.

 The present paper references other studies suggesting using SPT in the study of ethical consumption as well as other studies which suggest combining SPT and MLP, however, we miss a more thorough discussion with this literature which is referenced. Specifically, we will suggest that the paper includes the following discussions:

1. First, part of the literature already referenced in the paper has suggested to stay within SPT and from there find ways of including more overarching phenomena, including using the concept of Teleoaffective Formations (Welch 2020), the concept of General Understandings (Gram-Hanssen 2021) or discussions of how SPT relates to wider economic process (Evans, 2020). We think the paper would be highly improved if the authors went more into dialogue with these papers and their different attempts to close the gaps in SPT, before arguing for a move to include MLP.
2. Second, many authors have before this paper sought to combine SPT and MLP, as the authors of this paper also acknowledge. However, we miss a more thorough discussion on what is achieved by this combination and which problems it raises. The paper by Keller *et al.* 2022, has some relevant discussions on this, which we think this paper should go more into dialogue with.
3. Third, specifically (and related to the Keller *et al.* paper) a discussion on combining MLP and SPT with each other needs to be included, as most of the leading authors of SPT would argue that SPT is a flat ontology which is not the case with the levels of MLP. This is in our view not an argument for not discussing the gains of combining the approaches, however, the authors need to go into this discussion, at least to show they are aware of it, and how they suggest to overcome it, or work with it.

Is the argument persuasive and supported by appropriate evidence?

Partly

Is the work clearly and cogently presented?

Yes

Is the topic of the essay discussed accurately in the context of the current literature?

Partly

Does the essay contribute to the cultural, historical, social understanding of the field?

Yes

Reviewer Expertise:

consumption and theories of practice

We confirm that we have read this submission and believe that we have an appropriate level of expertise to confirm that it is of an acceptable scientific standard, however we have significant reservations, as outlined above.

# Response

**Published:** 2022-11-29

**Authors:** Sara Karimzadeh

**Affiliations:** Örebro University, Sweden

Dear Kirsten and Line   Thank you very much for your constructive comments. In the following, we briefly describe changes that we have made in the paper according to your comments.

  Thank you for this opportunity to review an interesting and timely contribution on the theoretical foundation for understanding ethical consumption. As the paper writes, theories of practice have been utilized for understanding consumption broadly, and more recently also specifically for understanding ethical consumption. However, as the paper also points out, most consumer studies so far based on theories of practice have been reluctant in studying overarching cultures and structures of consumer practices. For this reason, this paper suggests combining theories of practice, or social practice theory (SPT) with multi-level perspectives (MLP) theories, which we agree is a relevant approach to take.

 The present paper references other studies suggesting using SPT in the study of ethical consumption as well as other studies which suggest combining SPT and MLP, however, we miss a more thorough discussion with this literature which is referenced. Specifically, we will suggest that the paper includes the following discussions:

- First, part of the literature already referenced in the paper has suggested to stay within SPT and from there find ways of including more overarching phenomena, including using the concept of Teleoaffective Formations (Welch 2020), the concept of General Understandings (Gram-Hanssen 2021) or discussions of how SPT relates to wider economic process (Evans, 2020). We think the paper would be highly improved if the authors went more into dialogue with these papers and their different attempts to close the gaps in SPT, before arguing for a move to include MLP.

  Response: We agree. In response to this comment, in section two and paragraph three, we explained recent developments in social practice theory through the mentioned articles/concepts in the comment. We then argued that a multilevel understanding can progress our understanding of the phenomenon.

  

- Second, many authors have before this paper sought to combine SPT and MLP, as the authors of this paper also acknowledge. However, we miss a more thorough discussion on what is achieved by this combination and which problems it raises. The paper by Keller *et al.* 2022, has some relevant discussions on this, which we think this paper should go more into dialogue with.

  Response: We agree Keller et al. have several relevant points, which we could build more on. In response to this comment, we tried to strengthen our argument and the main changes appear in sections one, second and four.

  

- Third, specifically (and related to the Keller *et al.* paper) a discussion on combining MLP and SPT with each other needs to be included, as most of the leading authors of SPT would argue that SPT is a flat ontology which is not the case with the levels of MLP. This is in our view not an argument for not discussing the gains of combining the approaches, however, the authors need to go into this discussion, at least to show they are aware of it, and how they suggest to overcome it, or work with it.

  Response: We have now explicitly commented on the difference between flat and vertical ontology in section two.  
